# Gram positive bacterial infections in immunocompromised patients with HIV infection

**DOI:** 10.1186/1471-2334-13-S1-P30

**Published:** 2013-12-16

**Authors:** Andreea Cristina Stoian, Florentina Dumitrescu, Irina Niculescu, Cristina Iocu, Liliana Marinescu, Mariana Stănescu, Oana Bădescu, Georgeta Rodica Stoian

**Affiliations:** 1 University of Medicine and Pharmacy Craiova, Romania; 2 “Victor Babeş” Clinical Hospital of Infectious Diseases and Pneumology, Craiova, Romania; 3 “Dr. Vlăescu” Medical Center, Craiova, Romania

## Background

We aimed to perform microbiological analysis of Gram positive infections in patients infected with HIV (PIH).

## Methods

We performed a retrospective and compared study, between 01 January 2007 – 31 December 2010, in the Infectious Diseases Hospital of Craiova, on two groups of adults patients (Px): group A - 114 PIH and group B - 1442 Px non HIV, which had been diagnosed with non-tuberculosis bacterial infections (BI) with established etiology. For each group we analyzed the incidence, clinical spectrum, etiology and antibiotic susceptibility phenotype of Gram positive infections (GPI). For the statistical analysis we used the Fischer test; threshold values are statistically significant for p<0.05 and relative risk (RR) >2.

## Results

Incidence: group A vs. group B: BI-114 PIH (22.66%) vs. 1442 Px (8.7%) (p=0.000, RR=17.23), GPI-59 (51.75%) vs. 378 (26.21%) (p<0.0001); *S aureus* – 31 PIH (27.19%) vs. 58 Px (4.92%) (p=0.000; RR=6.42), *S pneumoniae* – 24 PIH (21.05%) vs. 234 Px (16.22%) (p=0.01; RR=2.79), *Enterococcus* spp. – 2 PIH (1.75%) vs. 7 Px (0.49%) (p<0.05), *S pyogenes* – 2 PIH (1.75%) vs. 77 Px (5.33%) (p<0.05) and *S viridans* – 2 Px (0.14%) only in group B. The clinical spectrum was dominated by lower respiratory tract infections: 29 PIH (49.15%) vs. 237 Px (69.09%) (p=0.004). Sensitivity to antibiotics: *S pneumoniae*, group A vs. group B: teicoplanin, linezolid 24 (100%) vs. 234 (100%) (p<0.05), moxifloxacin, 24 (100%) vs. 228 (97.43%) (p<0.05), vancomycin 24 (100%) vs. 228 (97.43%) (p<0.05), erythromycin -18 (75%) vs. 166 (70.94%) (p<0.05), co-trimoxazole 9 (37.5%) vs. 119 (50.84%) (p<0.05), penicillin 10 (41.62%) vs. 92 (39.31%) (p <0.05), ceftriaxone, 21 (87.5%) vs. 198 (84.61%) (p <0.05), rifampicin 18 (75%) vs. 217 (92.73%) (p=0.000) antibiotic sensitivity of *S aureus*, group A vs. group B (41 strains tested): oxacillin 24 (77.41%) vs. 33 (80.48%) (p>0.05) erythromycin, 13 (41.93%) vs. 21 (51.21%) (p<0.05), co-trimoxazole 8 (25.80%) vs. 29 (70.73%) (p<0.05), amikacin 29 (93.54%) vs. 38 (92.68%) (p<0.05), vancomycin 31 (100%) vs. 39 (95.12%) (p<0.05), linezolid 31 (100%) vs. 41 (100%), clindamycin 29 (93.54%) vs. 38 (92.68%) (p<0.05).

## Conclusion

Gram positive infections are more common in PIH versus Px non-HIV; the clinical spectrum is dominated by acute respiratory tract infections, regardless of HIV status. HIV infection was associated with high risk of resistance of *S pneumoniae* to co-trimoxazole, rifampicin and of *S aureus* to co-trimoxazole.

